# Medical Items and News of Interest to the Physician

**Published:** 1873-07

**Authors:** 


### Medical Items and News,

### For the Use of Physicians.

CULLED FROM THE STANDARD MEDICAL JOURNALS OF THE WORLD.

NOTE.—These formulæ are intended only for the regular practitioner of medicine, and should be employed only by him, or under his immediate supervision.

**Erethema, or Simple Inflammation of the Skin.**

One grain of sulph. atropia rubbed up with an ounce of glycerine forms an excellent application to inflammations of this character.

**Ringworm.**

The application of the pure, fresh citrine ointment (ung. hydr. nit.) seldom fails to cure this disease. Apply it twice a day, and keep the part clean.

**Eczema.**

The citrine ointment diluted with olive oil, 1 to 2 or 4, to which two grains of morphia to the ounce is added, often affords a cure or prompt relief in this perplexing disease.

**Iodine of Potassium in Bright's Disease.**

Professor Cryni, of Brussels, and others, have found favorable results from the administration of large doses of this salt in the second stages of Bright's Disease.

**Nervous Sick Headache.**

One fourth of a grain of ipecac, repeated every half hour or hour, has relieved many cases of this affection, and if the ipecac is continued in one to three grain doses, three or four times daily, a cure will frequently result—at least the intervals will be prolonged.

**Laxative Powder of Jeannel.**

Take of Rochelle salts 50 parts.
White sugar 100 parts.
Bicarbonate of Soda 22 parts.
Tartaric acid, powdered 20 parts.
Oil of lemon q.s.

Mix.—Dose, a teaspoonful in sweetened water.

**Sulphate of Iron in Erysipelas.**

This affection is said to be rapidly relieved by washing with a lotion composed of—

Sulphate of iron ʒj;

Water ℥vj.

It speedily relieves itching, heat, redness, and swelling. For poisoning by rhus toxicodendron, it is equally useful.

**Whitlow.**

On the first approach of this painful affection, if the point over the painful part be painted with pure deliquescent carbolic acid undiluted, the farther progress of the inflammation may be prevented, especially if we apply a coating of collodion over this.—*Medical Archives.*

**Elongation of Uvula.**

Dr. F. B. A. Lewis, in the *Boston Medical & Surg. Jour.*, records the case of a farmer, æt. 40, in whom the snipping off of one fourth of an inch of the elongated, uvula gave immediate relief to a distressing irritative cough, which had interfered with his sleep and greatly impaired his general condition.

**Sciatica.**

Some cases of this disease which has resisted a variety of treatment, were cured at Bellevue Hospital, *almost at once*, by the hypodermic injection of morphia over the seat of pain, plunging the needle deep into the tissues, perhaps to the depth of one or one and a half inches.—*N. Y. Med. Record.*

**Sal Ammonica in Dropsy.**

*The Clinic* states that Dr. Spencer Thomas, of Torquay, finds chloride of ammonium, or sal ammonica, one of the most efficient remedies in cases of portal dropsy, prescribed in scruple or half-drachm doses, given tolerably diluted, every six of eight hours. The recent accounts of the success attending the employment of this remedy in hepatic affection in India, he hopes will lead to its more extensive use in England.

**Carbolic Acid as a Local Anæsthetic in Boils, etc.**

Dr. J. N. Taylor, of Lynchburg, Tenn., writes as follows to the *Nashville Journal of Medicine*:

"About the middle of November, I was the subject of a very large and painful boil, on the ulna side of the forearm. I saturated a piece of worn linen in an aqueous solution of carbolic acid, and applied until all sensibility of the part had ceased; then, with a piece of ordinary writing-paper, twisted to a sharp point, and dipped into a solution of the acid (full strength), marked out the line of the desired incision; then, with an ordinary scalpel, I made a free incision, fully an inch in length, without any pain whatever.

"I greatly prefer the application of the acid, thus used, to the freezing process."

**Exophthalmic Goitre.**

J. Forsyth Meigs, M. D., Physician to Pennsylvania Hospital, (*Phila. Med. Times,*) in his clinical lecture "On a case of Exophthalmic Goitre," says that, in his later cases, he has used digitalis with benefit. Digitaline granules, gr. 1-60, three times a day, sometimes answered perfectly.

**External application of Veratrum-Viride.**

Dr. Anson Ford, of Buttle City, Montana, (*Jour. Gynæcological Soc.*), writes, that in the absence of all the remedies usually exhibited, he applied to a *supposed cancer of the left breast* Norwood's tincture of veratrum-viride, and was astonished at its immediate and almost magical effects. The induration of the gland and the excruciating pain disappeared under this treatment. A cloth was kept over the gland, saturated with the solution, and over all oil silk, to prevent evaporation.

**Sweating and Belladona.**

In *The Practitioner*, Dr. Sidney Ringer mentions the fact that belladona and its active principle are able to check and prevent sweating, whether the result of disease or induced by exposure to an elevated temperature. In the former case his observations enabled him to conclude that one-hundredth of a grain of atrophia injected under the skin is generally sufficient to check sweating for one night.—This dose produces dryness of the fauces, but does not dilate the pupils. Stramonium, it was found, is able to exert the same influence.

**Warm Salt Baths in Infantile Fevers.**

The difficulties which attend the use of cold baths, when it is desirable to reduce the temperature in very young children, has led Schwalbe, of Zurich, to try warm baths. His patient was a delicate rachitic child suffering from catarrhal pneumonia, and but little over a year old. The baths contain from 3 to 5 per cent. of common salt, and were given at a temperature varying between 80° and 88° Fahrenheit. At first they were employed from once to three times daily. The child made a good recovery. Many months after a similar attack occurred, and the same treatment was adopted, with the exception only, that the baths were more frequently repeated. The child made a favorable recovery the second time. It was observed that after each bath the temperature of the body was reduced from 2 to 4 degrees.

**Chronic Eczema.**

Dr. MacCallum, of the Montreal Gen'l Hospital gives the following mode of treatment: A solution of Potassa Caustica gr. v, to water ℥ ij, was used locally; brushed lightly over the eruption once daily. The following mixture is given internally:

℞

Hydrarg. bichlor. gr. iv;

Liq. Arsenic, ♏ xv;

Acid Hydrochlor. dil. ʒ iss;

Aquæ ad ℥ viij. M.

Sig:—℥ ss ter die.

**Cerebro Spinal Menengitis.**

Prof. Loomis, of New York, who has had considerable experience in the treatment of this affection, gives the following:

Sol. saturat. potass. bromid., forty minims every two or three hours; quiniæ sulph., three to five grains, every three hours; ice to the head and spine; blisters to the nape of the neck; bleeding, when the constitution of the patient will admit of it, and tonics during convalescence.—*Boston Med. and Surg. Jour.*

**Vienna Exhibition.**

A system of sanitary service is organized in Vienna, for the purpose of giving immediate attention to accidents, and carrying out measures of public hygeine, during the International Exhibition. There will be five stations, at each of which physicians will attend daily in town; and at each will be provided dispensaries, together with a military carriage, for the transport of persons severely injured. At the central station there will be a small hospital for the temporary reception of patients while waiting for the means of conveyance.—*London Med. Record.*

**Pneumonia.**

The physicians of Charity Hospital, New York, (*Medical Record*), are in the habit of prescribing about 15 grains of quinia per day, in 5 grain doses, and 6 grains of carbonate of ammonia every two hours. The oil-silk jacket is looked upon favorably, especially when bronchitis is present. The quinine is given for its tonic and anti-cell producing effect, and the carbonate of ammonia, not so much to retain the fibrin in a fluid condition as for its stimulant effect. Alimentation is to be carefully attended to and the patients are to be well nourished. With this plan of treatment the report is that the patients do remarkably well.

**Neuralgia.**

One of the best methods of treating neuralgia is that recommended by Frosseau. A tight top thimble is filled with cotton wool, and a few drops of strong aqua ammonia dropped on it. The open mouth of the thimble is then applied over the seat of pain for a minute or two, until the skin is blistered. The skin is then rubbed off, and upon the denuded surface a small quantity of morphia (gr. ¼) is applied. This affords almost instant relief. A second application of the morphia, if required, is to be preceded by first rubbing off the new formation that has sprung up over the former blistered surface.

---

**Prurigo Treated with Iodoform.**

Prof. Tanturri, of Naples, recommends for the obstinate itching diseases of the skin known as prurigo an ointment of iodoform. This remedy (iodoform) has strong anæsthetic properties, so that it is efficacious by deadening the sensibility of the nerves of the surface. Iodoform contains about 90 p. c. of iodine. When piles are very painful, especially during defecation, such ointment would be found to possess great virtue. Moutre's formula for suppositories is—

Iodoform, powdered gr. xx;
Cocoa butter ʒ j.
Melt and mix for suppositories.

For friction of the skin, the ointment is made of the strength of one drachm of the iodoform to an ounce of simple ointment.

---

**Hæmoptysis.**

The House Physician of Charity Hospital, (*Medical Record*), when the patient has a sudden attack of hæmoptysis, makes a local application of ice or cold sponges over the surface of the chest. In these troublesome cases, where blood is expectorated, more or less in quantity, every two or three days, or once a week and so on, some of the patients have tr. ergot in half drachm doses three times a day, and in some instances they have received marked benefit for the use of this remedy.

Inhalations of balsam of tolu and sesquichloride of iron. The quantity usually employed being:

℞

Balsam tolu, ʒ j;

Sesquichloride of iron, ℈ ij;

Water, O. j.

In that class of cases to which reference has been made, these inhalations allay the irritation, and act in a very beneficial manner.

**Diarrhœa in Teething.**

In a clinical lecture "on the Primary Dentition of Children," by Francis Minot, M. D., (*Boston Med. & Surg. Jour.*), in speaking of the diarrhœa complicating teething during hot weather, recommends the common chalk mixture with the addition of one fourth part of tincture of kino, which increases its astringency, and also keeps it from turning sour in hot weather. If the diarrhœa be not checked by this mixture, one drop of laudanum may be added to a dose, but not oftener than three times a day, to children under two years old. Diarrhœa is most apt to attack children who are brought up on the bottle; hence, if the case be urgent, and do not yield to treatment, a wet-nurse should be procured if possible.—When this cannot be done, he would strongly recommend the method of preparing the milk with arrow-root and gelatine, found in the treatise on "Diseases of Children," by Drs. Feigo and Pepper. Brandy is very useful to a teething child exhausted by diarrhœa, which should be given once in three or four hours, or oftener in urgent cases. The dose is ordinarily from five to twenty-five drops, in milk; but if there be much prostration the physician need not fear to increase the amount.

---

**Fluid Ext. Chestnut Leaves in Pertussis.**

Thos. D. Davis, M. D., of Dayton Ohio, (*Phila. Medical Times*), speaks of an epidemic of pertussis which broke out in the winter of 1870, while a resident physician in the Phila. Children's Asylum, in which this fluid extract was successfully used. Dr. Davis says that Mr. Maisch, of Phila., is the only druggist there who has a reliable preparation of this article. The leaves are gathered from July to October, the preference being given to those procured late in the season. The following is Mr. Maisch's formula for making the fluid extract:

Chestnut leaves, dried, cut and bruised, sixteen ounces; glycerine, five ounces; sugar, eight ounces; and hot water, a sufficient quantity; the extract to measure sixteen fluid ounces. The dose is half a teaspoonful to a teaspoonful every three or four hours, for a child six years old.

In urging this remedy upon the notice of the profession, he does not claim for it a specific action, but thinks it will be found a valuable anti-spasmodic and expectorant, the preparations of which are not unpleasant, and are readily taken by children.

**An Ointment for Neuralgia.**

Dr. J. Knox Hodge recommends the following as an application which will relieve facial or any other neuralgia almost instantaneously :

℞

Albumen of egg ʒj.

Rhigolene ℥ iv.

Oil of peppermint ℥ ij.

Collodion; chloroform āā ℥j. M.

Agitate occasionally for twenty-four hours, and by gelatinization a beautiful semi-solidified opodeldoc-looking compound results, which will retain its consistency and hold the ingredients intimately blended for months.—Apply by smart friction with the hand, or gently with a soft brush or mop along the course of the nerve involved.

---

**Bronchitis.**

Dr. J. A. Lidell writes to the New York *Medical Record* as follows :—

In a bad case of chronic bronchitis—a case in which there was strongly marked bronchiectasis on both sides, harassing cough both by day and night, profuse muco-purulent secretion that oftentimes was very offensive in smell, and emaciation with other general signs of bronchital phthisis—the writer has recently administered carbolic acid by inhalation, and made the patient comfortable by so doing, when every other palliative had failed.

At first it was given in the vapor of hot or warm water; but, after a short trial, these inhalations were discontinued, because they made the patient perspire too much. Then it was administered in the form of spray with Codman and Shurtleff's atomizing apparatus No. 5, and the result was gratifying in every respect. The preparation which was used mostly consisted of the crystallized acid dissolved in water in the ratio of one grain of the former to one ounce of the latter; that is, 1 part of the acid to 480 parts of water. Trials were also made with a solution as weak as 1 part to 600, on the one hand, and as strong as 1 part in 200, on the other, but those having a strength of 1 part to 450 or 480 answered best. The patient was made to breathe or inhale the spray with deep inspiration, from five to ten minutes at a sitting, unless a feeling of drowsiness were sooner produced, once a day usually; twice a day, however, when the expectoration was very profuse or offensive in smell.

**Varicose Ulcers.**

Dr. A. J. Steele, of St. Louis (*Med. Archives*), speaks with confidence in the treatment in these ulcers, of that method first proposed by Mr. John Scott, formerly surgeon to St. George's Hospital, after an uniform success in twenty-five cases. The plan, though modified in detail, still retains the author's central idea, which was—equable support to the vessels of the entire foot and leg, and especially of all that part below the ulcer.—Nothing accomplishes this end so efficiently as the properly applied adhesive plaster. Bandages alone have not the firmness; they loosen and become relaxed. It is essential that the plaster be evenly applied—no folds or creases, no uncovered angles or spaces—otherwise there will result irritation, pain excoriation. After the strapping he applies a flannel bandage, from five to seven yards in length, and about two inches in breadth. The entire foot and leg to the knee should be included.

---

**Internal Piles.**

Billroth records 26 cases of prolapsing piles treated by him in various ways. In four instances he applied the actual cautery, in ten the galvano-cautery, and in the remainder fuming nitric acid. The latter plan was pursued as recommended by Dr. Houston, of Dublin. The results proved eminently satisfactory. His mode of proceeding was as follows: A free evacuation of the bowel was obtained by means of castor-oil given the day previously. Before the operation the mass was brought down by an injection. The patient was then placed on the side with the knees flexed. The parts adjacent to the anus were first well protected by oil so that no injury should be done them. A small piece of wood was then dipped in the acid and applied to the outside of the swollen mass, until it had become tolerably stiff and had assumed a yellowish-green color. It was then smeared with some simple form of ointment, and returned within the sphincter. The operation was usually performed without an anæsthetic, an opiate suppository was rarely given afterwards. It is proper to keep the patient in bed. Fever rarely follows, though retention of urine is not uncommon for the first few days. The eschar usually separates without loss of blood. It is proper to give castor-oil on the third or fourth day, provided no fæces have passed. Hemorrhage will likely occur if the fæces become hardened; such accidents are readily controlled by ice. Of the patients treated in this manner, some were discharged on the 5th and 6th days, though severe cases were under treatment from six to eight weeks.— Several of the patients were examined a year after the operation, and there was no stricture in any one of them. Billroth believes that in very severe cases this treatment will fail, and then suggests the use of the acid nitrate of mercury, as recommended by Curling.— *Weiner Med. Wochen.*

---

**Fissure of the Nipple.**

In a paper by Dr. Criquey, fissures of the nipple are described as being of two kinds: First, those produced by the violent suction of the child; here the epidermis is raised and abraded, as if by a cupping-glass. In this condition of the nipple, the child should be allowed to suckle only when the breast is charged with milk. Second, and at other times, a little of the milk lodges in the minute cracks at the base of the nipple, where it comes in contact with the secretions of the body and rapidly decomposes, thus acting as an active irritant of the skin, and in some instances inducing very extensive inflammation. As a preventive of cracked nipples, originating in this manner, the breast should be bathed in warm water, wiped dry, and then annointed with the following ointment:

℞

Tannin, 1 gramme;

Glycerine, 10 grammes.

This should be applied by means of a camel's hair brush, after which the nipple should be protected with charpie, or a soft linen cloth. In these cases the nipple shield may be employed to advantage. If the breast be distended with milk, relief may be afforded by the application of a large flaxseed poultice, taking the precaution to protect the nipple with a piece of soft leather--*Gaz. des Hop.*

---

**Treatment of Acute Rheumatism by Propylamine and Trymethylamine.**

Propylamine was first employed by Awenarius, of St. Petersburg (*Journal de Physique et de Chimie*, 3e serie, t. xxv., 1859), with success in more than 250 more or less complicated cases of acute rheumatism. Upon his recommendation Dr. Gaston (*Medical Press and Circular*, 1872) used this remedy, and asserts that it will produce decided improvement even in severe cases in thirty-six or forty-eight hours. Dr. Gaston, however, employed quinia with propylamine. M. Dunjardin-Beaumetz (*L' Union Medicale*, Jan. 16, 1873) reports seven cases, six of acute, and one of chronic rheumatism, treated with propylamine alone. Six of these cases recovered between the third and tenth day of treatment. In very acute cases, where the remedy was employed at the commencement of the attack, the results were remarkably rapid.— In twenty-four to forty-eight hours at most the pain disappears, then the redness and swelling diminish, and at the same time the fever declines; relapses are slight and of short duration. From these few cases (some of which had cardiac disease at the time of their admission into hospital), he is unable to speak of the influence of propylamine on the heart complication. He commences with a dose of 0.5 gramme, increasing to 1 gramme, and even to 1.5 gramme. This does not disturb the stomach. Dr. A. Besnier reports six cases of rheumatism treated in this way with decided success.—*Am. Jour. of Med. Sciences.*

---

**Treatment of Diabetes Mellitus.**

Professors Cantani and Primavera, of Naples, report the most extraordinary success in their treatment of this obstinate disease. Their statements are in brief as follows:—

1. Their patients have all, with rare exceptions, recovered.

2. Stout persons have lost but little weight during the treatment, while spare ones have sometimes gained as much as twenty-five pounds.

3. Though the urine has become rich in urea and uric acid, the patients have never shown symptoms of gout or urinary calculi.

4. The treatment was also successful in arresting some instances of albuminuria that accompanied the disease.

5. The cure consists in an exclusive meat diet, and by this term fish is also included; further, at each meal is to be taken lactic acid ℈ ij--iv in water ℥ vj. As a substitute for wine at dinner, alcohol ℥ ss. with water ℥ vj is given.

Alcohol and lactic acid are designed to replace the saccharine and starchy elements of the food. To obtain a permanent cure it is necessary to persist in the treatment for several months after sugar has ceased in the urine, Then the patient may gradually return to a mixed diet.—*Allgemein. Med. Central. Zeitung.*

**Phthisis.**

The general therapeutics in these cases are such as the profession are already familiar with. The only new remedy which has been employed for the relief of the night-sweats is the tr. of belladonna, given in ordinary doses, once or twice a day. This remedy has given very satisfactory results in all cases in which it has been used. For the purpose of subduing the fever, which in some cases becomes the most prominent symptom for treatment, Heim's pill has been used with excellent results:

℞

Pulv. herb. digitalis, ℈ ss;
Pulv. rad. ipecac.,
Pulv. opii puri, āā grs. v;
Ext. helenii, q. s. u. f. pill. No. xx.

Consp. pulv. rad. ind. flor.

Sig: A pill three times daily.

To control the diarrhœa which sometimes appears, salicine has been used with better success than any other remedy which has been employed. It is given in ten grain doses every four hours, either in the form of pill or powder, as most agreeable to the patient. Dr. Drake, House Physician to Charity Hospital, who first introduced it into the Hospital, has treated seven cases by the use of salicine, which has resisted every form of treatment which had been adopted, and cured every one of them. What is equally satisfactory is, they have remained cured. Salicine has therefore become to be one of the methods of treatment for chronic diarrhœa, whatever cause it may be dependent upon, or with whatever disease it may be associated. There are occasional cases which do not respond to this remedy; but in general, the results have been far more satisfactory than with the use of any other remedy.—*Medical Record.*

---

**Bellevue Hospital.**

The following notes of practice in this institution have been published in the *Medical Record:*——

Ulcers.—The old, chronic ulcers, with indurated edges, are sometimes immensely benefitted by making numerous free incisions through their indurated tissues, even down to and through the periostium, if that structure is thickened. This is followed by a firm strapping with adhesive plaster. The result is a reduction of the congestion which has long existed in the parts, and the production of an alterative action in the tissues, which renders them more healthy. Some wonderfully obstinate cases are cured by this method of treatment.

Drop-Wrist.—A cure of "drop-wrist" has been under treatment by the use of galvanism and hypodermic injections of strychnia—1-75 of a grain of the sulphate twice a day. This patient had received various kinds of treatment before his admission to the hospital, but without benefit. The ordinary iodide of potassium treatment has given him no relief whatever, but from the date of the commencement of the treatment by the use of the strychnia and galvanism, he began to improve rapidly.

Tape Worm.—Without doubt the success of any remedy depends very much upon the method in which it is administered. A patient here has been treated in the following manner with success. The remedy used was the Male Fern:

℞

Liquid Extract Male Fern,
Syrp. Gum Arabic, āā ℥ ij. M.

The patient took nothing for supper, and in the evening took a full dose of castor-oil. In the morning she took no breakfast, but took a desert spoonful of the mixture, and repeated it every four hours until an effect was produced upon the bowels, or the stomach ceased to tolerate it. In general, the remedies for the removal of this parasite are to be taken fasting; and, doubtless in many cases success has been denied on account of a want of specific directions in regard to the manner of using the remedy employed.

Nitrate of Amyl.—This is a remedy which is used somewhat for the relief of dyspnœa. An old lady, the subject of ephysema and chronic bronchitis, has her vial of amyl upon her stand, and when she feels her attacks of dyspnœa coming on, she allays them very readily by dropping a few drops on some lint —5 is the usual number directed—and inhaling it. It is of benefit in the dyspnœa of bronchnial asthma, the dyspnœa of that peculiar grouping of symptoms which go to make up angina pectoras, and dyspnœa from any similar cause.

Burns.—The remedies used in the treatment of this class of accidents are as numerous as the visiting surgeons and the house surgeons are disposed to devise. Common white paint kept continually spread upon the surface is the remedy commonly used. Equal parts of Garland's Citrate and sweet oil is a remedy favorably known. A very satisfactory remedy is "Dr. Buck's Burn Mixture." The following is the formula for its preparation :

℞ Gum Tragacanth. ℥ij ;
Gum Acacia, ℥iv ;
Molasses ;
Aqua, āā ℥j.

Mix the gum and water and let them remain until thoroughly dissolved, and then add the molasses. This is spread over the surface with a brush, forming a continuous coating, and if removed by the process of suppuration, it is to be immediately reapplied. Syrp. Acacia with sufficient Glycerine to make a liquid which can easily be spread, is sometimes used and then covered with lycopodium.

Sprains.—The immovable apparatus is usually applied at once. Immersing the limb in hot water, permitting it to remain some time, and then applying a snug roller, is a most excellent method of treatment.

Erysipelas.—A single case of erysipelas attacking an injured limb was treated by the immersion plan. The limb was kept immersed in luke-warm water constantly for ten days, with exceedingly gratifying results. Whether this method of treatment will be retained or not, can only be determined by further use.

Sub-Acute Rheumatism.—For the relief of the stiffness which remains about the joints after an acute attack, the oleate of Mercury and Morphia is somewhat employed, and with very gratifying results. The attention of the profession has been called to this compound by its author, in an article published in the last number of *Braithwait's Retrospect*, where a more complete description can be found.—Ringer also mentions it in his work upon therapeutics.

Prepared Earth.—This material is used in dressing surfaces which have a gangrenous offensive discharge ; like the sloughy surfaces following frost-bites. The earth is spread upon a cloth and wrapped about the sore, and will remain 24 hours without becoming very offensive. The material is specially prepared at Philadelphia, and can be obtained for the nominal sum of $3 per barrel. I suppose that any clayey earth, perfectly dried and thoroughly pulverized, will answer the purpose equally well.

